# Patient‐side performance metrics and errors in robotic surgery: Junior‐ European Association of Urology Robotic Urology Section/Young Academic Urologists (J‐ERUS/YAU) construct validation

**DOI:** 10.1111/bju.70199

**Published:** 2026-02-25

**Authors:** Christoph Würnschimmel, Malte Boese, Razvan Ognean, Marco Paciotti, Mike Wenzel, Carlo Andrea Bravi, Ruben De Groote, Paolo Dell'Oglio, Fabrizio Di Maida, Marcio Covas Moschovas, Federico Piramide, Filippo Turri, Gabriele Sorce, Nikolaos Liakos, Anthony Gallagher, Domenico Veneziano, Ton Brouwers, Markus Graefen, Evangelos Liatsikos, Alberto Breda, Alessandro Larcher, Iulia Andras

**Affiliations:** ^1^ University Research and Teaching Hospital Lucerne Switzerland; ^2^ Faculty of Health Sciences and Medicine University of Lucerne Lucerne Switzerland; ^3^ Martini‐Klinik Prostate Cancer Center University Hospital Hamburg‐Eppendorf Hamburg Germany; ^4^ Department of Urology University Hospital Frankfurt, Goethe University Frankfurt Frankfurt am Main Germany; ^5^ Department of Urology, Faculty of Medicine Medical Centre of the University of Freiburg Freiburg Germany; ^6^ Department of Urology Iuliu Hatieganu University of Medicine and Pharmacy Cluj‐Napoca Romania; ^7^ Department of Urology IRCCS Humanitas Research Hospital Rozzano Milan Italy; ^8^ Department of Urology ASST Niguarda Hospital Milan Italy; ^9^ Department of Urology, ASST Santi Paolo e Carlo University of Milan Milan Italy; ^10^ Division of Experimental Oncology, Department of Urology, Urological Research Institute (URI) IRCCS San Raffaele Scientific Institute Milan Italy; ^11^ Unit of Oncologic Minimally Invasive Urology and Andrology, Department of Experimental and Clinical Medicine, Careggi Hospital University of Florence Florence Italy; ^12^ Division of Urology, Department of Oncology, San Luigi Gonzaga Hospital University of Turin Orbassano Turin Italy; ^13^ Department of Urology IRCCS San Raffaele Scientific Institute Milan Lombardia Italy; ^14^ Department of Urology Northampton General Hospital Northampton UK; ^15^ Department of Urology The Royal Marsden NHS Foundation Trust London UK; ^16^ School of Medicine, Faculty of Life and Health Sciences Ulster University Belfast UK; ^17^ Department of Urology OLV Hospital Aalst Belgium; ^18^ ORSI Academy Melle Belgium; ^19^ Global Robotics Institute AdventHealth Celebration FL USA; ^20^ School of Medicine Hofstra Northwell University New York NY USA; ^21^ European Association of Urology Arnhem The Netherlands; ^22^ Department of Urology University of Patras Patras Greece; ^23^ Department of Urology, Fundació Puigvert Autonomous University of Barcelona Barcelona Spain

**Keywords:** proficiency‐based progression, patient‐side assistance, robot‐assisted radical prostatectomy, performance metrics, error, critical error, surgical education

**Abbreviations**
ERUS: European Association of Urology Robotic Urology Section
PA: patient‐side assistant
PBP: proficiency‐based progression
RARP: robot‐assisted radical prostatectomy
YAU: Young Academic Urologists

Proficiency‐based progression (PBP) training is a modern system to educate surgeons using a structured methodology, and includes essential performance metrics, as well as errors and critical errors to be avoided during surgery [1]. The European Association of Urology Robotic Urology Section (ERUS) and the Young Academic Urologists (YAU) Robotic Section initiated the development of PBP training curricula for robotic surgeons [2, 3]. Increasing attention has also been directed toward patient‐side assistants (PAs), who have historically been underrepresented in robotic training. This gap is notable, as the vast majority of PAs in a recent survey expressed a need for structured training, and growing evidence suggests that PA proficiency may directly influence surgical outcomes [4, 5]. Therefore, in order to create a PBP‐based PA training curriculum, we previously reported face‐ and content validity for novel, Delphi‐consensus based PA performance metrics, errors and critical errors. These metrics were fitted to be used for PBP‐based trainings in robot‐assisted radical prostatectomy as reference procedure [6]. Consequently, we aim to establish construct validity for these proposed performance metrics.

The previous Delphi‐based consensus [6] provided a concise list of performance metrics (see the open access supplementary table in the earlier publication), including common PA's errors and critical errors to be avoided during robot‐assisted radical prostatectomy (RARP). In short, this list tested performance during three critical phases that were defined in the previous delphi‐based consensus: (i) Docking, (ii) Intraoperative, and (iii) Undocking, and within these, errors and critical errors could be of technical but also non‐technical nature. Construct validation was assessed by experienced robotic surgeons in novice PAs and expert PAs, in three different tertiary care centres (Lucerne, Hamburg, and Cluj‐Napoca) by live‐assessment of proficiency using the previously established list of errors and critical errors. Experience was defined as a minimum of 250 RARPs as a PA and 50 RARPs as a console surgeon. Assessors at each centre conducted a dedicated explanatory meeting to review the performance metrics, errors, critical errors and standards for live assessment, ensuring inter‐centre comparability. We hypothesised that, after following PBP‐based instruction of PA metrics, error rates would drop in each round of assessment. After each surgery, the assessor provided the PA with the feedback of errors and critical errors observed in this assessment round, together with a qualitative discussion on potential improvements for the next round. Simultaneously, we assumed that expert PAs would exhibit low error rates already in first round of assessment. The PAs went through repeated assessments. Known‐groups validity of experts vs novices was assessed descriptively, while within‐novice learning effects (defined as median start to end change of total errors according to number of prior surgeries performed) were plotted using slopes for each novice PA.

We analysed 62 assessment rounds from 11 PAs (seven novices and four experts). In the expert group, in total, two errors and zero critical errors were detected during six assessment rounds. In the novice group, in total, 44 errors and nine critical errors were detected during 56 assessment rounds. Six of seven novices (86%) reduced total errors from first to last observation and exhibited negative slopes in terms of errors made during the procedure (median error reduction −0.37 per surgery, Fig. 1). As the expert comparison group exhibited low error counts (total two errors) from the very beginning, slopes were not calculated. No errors occurred in the repeated assessments of expert PAs. Novices most often erred during the intraoperative phase, while docking and undocking phases also showed gaps, but these were smaller and less frequent. More specifically, the most common errors made were ‘Failure to clip the correct designated structure as indicated by the console surgeon’ (18 counts), ‘Failure to ask the console surgeon for an overview when new robotic or laparoscopic instrument is inserted’ (11 counts), ‘Failure to clean the camera port prior to introduction of the camera’ (eight counts), and ‘Failure to confirm that energy cables are connected to the generator’ (seven counts). Furthermore, the most common critical errors made were ‘Failure to safely clip a structure: Clip is falling off because trainee does not close the clip fully, or does not use full length, or removes clip applied before having opened the branches’ (22 counts), ‘Failure to use both hands when introducing robotic instruments into the trocar’ (five counts), and ‘Failure to ask console surgeon for instrument overview before withdrawing it’ vs ‘Failure to remove or introduce instrument under direct vision’ vs ‘Failure to avoid collision between tissue and laparoscopic scissors’ (*ex aequo* four counts).

**Fig. 1:**
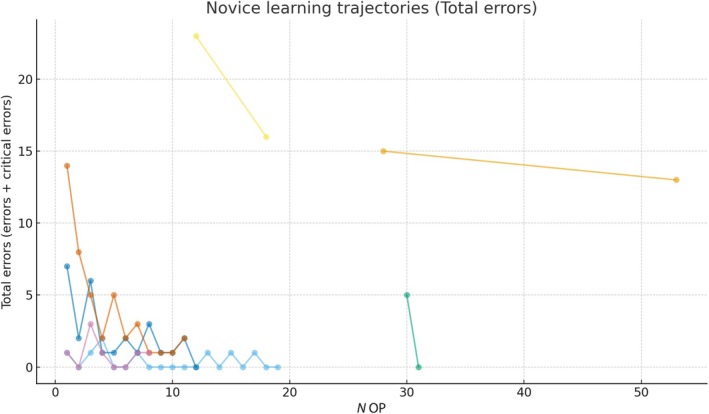
Total number of errors and critical errors performed by novice PAs during robot‐assisted radical prostatectomy in three tertiary referral centres during the evaluation phase of a Delphi‐based proposal of specific performance metrics. Each line represents a separate novice PA and exhibits the trajectory of total errors performed in repeated assessments. The *x*‐axis indicates the absolute experience in terms of previously assisted RARPs (number of operations [*N* OP]). More specifically, absolute experience ranged between a minimum of one previously assisted surgery in three novices, and 12, 28, 30, and 61 assisted surgeries in the remaining four PAs. The variation in the number of assessments per PA reflects real‐world logistical constraints, including differences in clinical scheduling, availability for live assessment, and progression through training during the study period, therefore assessments were performed opportunistically rather than at fixed intervals.

These findings extend our earlier face and content validation by showing that the same item set not only has expert/Delphi credibility but also exhibits expected performance patterns in the operating room, thus implying individual‐level construct validity for our proposed PBP‐based PA training curriculum. Furthermore, taking together all PAs performances, we observed consistent learning effects, with approximately one fewer error occurring every three surgeries. Using PBP, trainees can target the specific, discriminating errors and critical errors where novices most differ from experts. We critically identified a culmination of multiple errors and critical errors that should be avoided and propose several strategies: (I) Mandatory confirmation with the console surgeon before clipping, (II) dedicated and mandatory dry‐laboratory trainings of all instruments used prior to surgery, (III) Integration of verbal standard operating procedures for enhanced communication in terms of console surgeon and PA interaction, but also including the operating room personnel (e.g., correct plugging in of electrical cables and insufflators), and (IV) Video debriefings of critical errors. In future training curricula, these strategies can be adapted dynamically: new items can be added or refined as further experience accumulates, ensuring that the instrument evolves in parallel with the observed error patterns and educational needs. In terms of limitations, first, assessors were not blinded to the previous PA experience, and second, a small number of expert assessments were available while novice data were clustered through repeated measures. This combination, together with the lack of assessor blinding, may lead to conservative appearance of group contrasts. Finally, the analysis was limited to descriptive comparisons, and PAs were aware of being assessed, which may have introduced a Hawthorne bias. Importantly, these limitations do not undermine the core finding: novices consistently reduced errors with increasing experience, while experts maintained very low error counts (therefore, also slopes were not calculated for experts). This individual‐level construct validity, together with our prior face and content validation, provides strong evidence that this checklist is a robust educational tool. These results support its integration into PBP feedback curricula for robot‐assisted radical prostatectomy [7], which may now be followed up within dedicated courses for PAs led by the ERUS and YAU.

## Disclosure of Interests

The authors report no conflicts of interest for this manuscript.

## Funding

No funding was provided.
